# Mass Spectrometric Characterization of HSV-1 L-Particles From Human Dendritic Cells and BHK21 Cells and Analysis of Their Functional Role

**DOI:** 10.3389/fmicb.2020.01997

**Published:** 2020-09-29

**Authors:** Alexandra Birzer, Max Edmund Kraner, Christiane Silke Heilingloh, Petra Mühl-Zürbes, Jörg Hofmann, Alexander Steinkasserer, Linda Popella

**Affiliations:** ^1^Department of Immune Modulation, Universitätsklinikum Erlangen, Erlangen, Germany; ^2^Division of Biochemistry, Department of Biology, Friedrich-Alexander Universität Erlangen-Nürnberg, Erlangen, Germany; ^3^Department of Infectious Diseases, University Hospital Essen, University of Duisburg-Essen, Essen, Germany

**Keywords:** HSV-1, mass spectrometry, heavy particles, light particles, BHK21 cells, dendritic cells, T cell stimulation, immunomodulatory effect

## Abstract

Herpes simplex virus type 1 (HSV-1) is a very common human pathogenic virus among the world’s population. The lytic replication cycle of HSV-1 is, amongst others, characterized by a tripartite viral gene expression cascade, the assembly of nucleocapsids involving their subsequent nuclear egress, tegumentation, re-envelopment and the final release of progeny viral particles. During productive infection of a multitude of different cell types, HSV-1 generates not only infectious heavy (H-) particles, but also non-infectious light (L-) particles, lacking the capsid. In monocyte-derived mature dendritic cells (mDCs), HSV-1 causes a non-productive infection with the predominant release of L-particles. Until now, the generation and function of L-particles is not well understood, however, they are described as factors transferring viral components to the cellular microenvironment. To obtain deeper insights into the L-particle composition, we performed a mass-spectrometry-based analysis of L-particles derived from HSV-1-infected mDCs or BHK21 cells and H-particles from the latter one. In total, we detected 63 viral proteins in both H- and L-particle preparations derived from HSV-1-infected BHK21 cells. In L-particles from HSV-1-infected mDCs we identified 41 viral proteins which are differentially distributed compared to L-particles from BHK21 cells. In this study, we present data suggesting that L-particles modify mDCs and suppress their T cell stimulatory capacity. Due to the plethora of specific viral proteins incorporated into and transmitted by L-particles, it is tempting to speculate that L-particles manipulate non-infected bystander cells for the benefit of the virus.

## Introduction

The human pathogenic herpes simplex virus type 1 (HSV-1) represents the prototype of the α−herpesvirus subfamily. In contrast to the highly diverse and complex herpesvirus-associated diseases, herpesviruses share a conserved genome composition and virion morphology (Whitley and Griffiths, 2002; Schleiss, 2009; Watson et al., 2012). Common among all herpesviruses, HSV-1 consists of the viral DNA genome surrounded by a multi protein layer, known as tegument, and the outer glycoprotein rich host-derived membrane, forming the envelope (Laine et al., 2015).

After attachment to and penetrating the cell, viral tegument proteins play an essential role in modulating the infected cells for the benefit of the virus. Furthermore, specific tegument proteins enter the nucleus and support the initiation of the tripartite gene expression cascade divided into immediate-early (IE), early (E) and late (L) gene expression (Guo et al., 2010). In particular, the tegument proteins ICP0, ICP4, and VP16 are released into the cell and facilitate initiation of the gene expression cascade. Simultaneously, the virion host shutoff protein (vhs) destabilizes cellular mRNAs to, e.g., hamper the host’s immune response against HSV-1 (Taddeo et al., 2010; Dauber et al., 2014). After completion of the viral *de novo* protein synthesis, HSV-1 progeny capsids are assembled inside the nucleus and subsequently cross the nuclear membrane bilayer getting enveloped and de-enveloped at the inner nuclear membrane (INM, primary envelopment) and the outer nuclear membrane (ONM), respectively (Mettenleiter, 2002; Johnson and Baines, 2011; Crump, 2018). Primary envelopment and following de-envelopment in the perinuclear space requires the multiprotein nuclear egress complex (NEC), composed of the viral proteins UL31 and UL34 (Reynolds et al., 2002; Bigalke and Heldwein, 2017). Having passed the nuclear membrane, capsids get coated with tegument proteins by a step called tegumentation (Vittone et al., 2005; Henaff et al., 2013). In a last step, virions bud of cytoplasmic membranes, such as derived from the trans-Golgi network or endosomes, providing the lipid envelope of mature virions (secondary envelopment) for the subsequent release (Lv et al., 2019). Apart from mature infectious virions, so-called heavy (H-) particles, a lytic HSV-1 infection will also give rise to the production of light (L-) particles, which are void of the capsid and thus are not infectious (Hogue et al., 2016).

HSV-1 has established well elaborated strategies to efficiently infect and replicate in a variety of different cell types as well as several host species (Karasneh and Shukla, 2011). Apart from initially infected cells during a primary HSV-1 infection in, e.g., fibroblasts or epithelial cells, also immune cells, such as dendritic cells (DCs) can be infected (Smiley et al., 1985; Goldwich et al., 2011). DCs operate at the interface of the innate and adaptive immune system by presenting peripheral antigens to T cells for their activation, hence serving as promising targets for HSV-1-mediated immune modulations. In the last decades, several immune evasion mechanisms of HSV-1 regarding DC surface protein expression, migration, maturation and T cell stimulation have been deciphered (Kruse et al., 2000; Pollara et al., 2003; Prechtel et al., 2005; Theodoridis et al., 2011; Heilingloh et al., 2015).

Recent observations by Turan et al. revealed that HSV-1 exploits autophagic turnover to degrade nuclear lamins in immature DCs (iDCs), facilitating nuclear egress of viral capsids and thus virion assembly (Turan et al., 2019). By contrast to their immature counterparts, mature DCs (mDCs) inhibit efficient autophagic flux, and block autophagy-mediated lamin degradation upon HSV-1 infection. This in turn prevents HSV-1 nuclear egress and the formation of infectious virions, i.e., H-particles. However, during millions of years of co-evolution, HSV-1 evolved sophisticated strategies to bypass this dead end of replication. Intriguingly, during an HSV-1 infection of mDCs the virus produces non-infectious L-particles (Goldwich et al., 2011; Turan et al., 2019). While L-particles contain tegument proteins and the glycoprotein rich envelope (Szilágyi and Cunningham, 1991; McLauchlan and Rixon, 1992), these particles are characterized by the lack of the capsid and thus the viral genome. Moreover, in contrast to H-particles, L-particles are present in the cisternae of the rough endoplasmic reticulum (Alemañ et al., 2003; Hogue et al., 2016; Heilingloh and Krawczyk, 2017). Despite these prominent differences among H- and L-particles, both HSV-1-derived particle variants share similar maturation steps, especially in human neuronal cells (Granzow et al., 2001; Alemañ et al., 2003; Ibiricu et al., 2013). However, the biological function of HSV-1-derived L-particles during infection is yet not completely understood and thus requires further investigation to gain more insights into their role during HSV-1 replication and propagation. Concerning this, several authors previously proposed that the presence of L-particles can foster the infectivity of HSV-1 (McLauchlan et al., 1992; Dargan and Subak-Sharpe, 1997). Furthermore, mDC-derived L-particles are capable of modulating non-infected bystander mDCs via the transmission of viral proteins (Heilingloh et al., 2015). In particular, the functionally important glycoprotein CD83 is not only downregulated in directly infected but also in non-infected bystander mDCs via L-particles. Given their modulatory capacity, it seems reasonable to assume that L-particles possess a crucial role during HSV-1 infection.

Within the present study, we intended to get closer insights into the composition of L-particles derived from HSV-1-infected BHK21 cells and mDCs using mass spectrometry. We identified 63 viral proteins in mature infectious virions as well as in L-particles from HSV-1-infected BHK21 cells. Moreover, we report mass spectrometric analyses of L-particles derived from HSV-1-infected mDCs, whereby 41 viral proteins have been identified. Quantification of these proteins revealed that L-particles from the two different cell types possess a different protein composition. HSV-1-derived L-particles from BHK21 cells especially exhibit higher amounts of US2, UL31, UL34, and ICP8 compared to H-particles. By contrast, L-particles produced by mDCs upon HSV-1 infection are different to L-particles from BHK21 cells and are characterized by the high abundance of ICP6, ICP4, glycoprotein B (gB) and gD. Concerning the functional role of L-particles, our results suggest that L-particles modify CD83 surface expression during the DC maturation process as well as impair the T cell stimulatory capacity of mDCs. Due to the plethora of specific viral proteins incorporated into L-particles, we hypothesize that these non-infectious particles produced by HSV-1-infected mDCs have an immunomodulatory effect on DCs.

## Materials and Methods

### Generation of Dendritic Cells

For the generation of human monocyte-derived mature dendritic cells (mDCs) peripheral blood mononuclear cells (PBMCs) were isolated from leukoreduction system chamber (Pfeiffer et al., 2013). Lymphoprep solution was overlaid with the diluted blood (1:5 in PBS supplemented with 10% ACD-A) and centrifuged at 400 × *g* for 30 min at RT. The distinct intermediate phase was collected and washed three times with ice-cold PBS containing 1 mM EDTA. Subsequently, the PBMC pellet was resuspended in 10 mL of RPMI 1640. PBMCs (approximately 400 × 10^6^) were centrifuged at 300 × *g* for 5 min, resuspended in 25 mL DC medium (RPMI 1640 supplemented with 1% human Ab serum, 100 U/mL Penicillin, 100 U/mL Streptomycin, 2 mM L-glutamine and 10 mM HEPES) (all Lonza, Switzerland), transferred into T_175_ cell culture flasks and incubated at 37°C and 5% CO_2_ for 1 h. Cells were washed three times with pre-warmed RPMI 1640 (Lonza, Switzerland), and the non-adherent fraction was allowed to adhere in DC medium in an additional cell culture flask with subsequent discarding the non-adherent cells as described above. Adherent monocytes were cultivated in 30 mL of DC medium supplemented with 800 U/mL GM-CSF (Miltenyi Biotec, Germany) and 250 U/mL IL-4 (Miltenyi Biotec, Germany). On the fourth day, 5 mL of fresh DC medium supplemented with 400 U/mL GM-CSF and 250 U/mL IL-4 were added to each cell culture flask to generate iDCs. For the generation of mDCs, a maturation cocktail containing GM-CSF (40 U/mL), IL-4 (250 U/mL), IL-1ß (Cell Genix GmbH, Germany; 200 U/mL), IL-6 (Cell Genix GmbH, Germany; 1,000 U/mL), TNF-a (Peprotech, Germany; 10 ng/mL) and PGE2 (Pfizer, Germany; 1μg/mL) was added to each cell culture flask on day 5. Two days after induction of maturation mDCs were used for infection experiments. For the generation of L-particles, mDCs were used 1.5 days post maturation.

### Cells, Virus Strain and Amplification

HSV-1/17+/CMV-EGFP/UL43, herein designated as HSV-1, was obtained from the laboratory strain HSV-1 17+ via the insertion of a GFP expression cassette controlled by the CMV promoter into the UL43 locus [BioVex, (Coffin et al., 1996, 1998)]. This gene is not essential for HSV-1 replication and its deletion does not affect HSV-1 reactivation and latency (Mikloska et al., 2001). For virus amplification, 90% confluent BHK21 cells in T_175_ cell culture flasks were washed once with PBS and infected in 5 mL of infection medium (RPMI 1640 with 20 mM HEPES) containing a defined amount of HSV-1 virions (MOI of 0.01). The infection was performed on an orbital shaker at RT for 1 h. Subsequently, 20 mL of DMEM [supplemented with 10% FCS, 2 mM L-glutamine, 100 U/mL Penicillin, 100 U/mL Streptomycin and 1% non-essential amino acids (100× stock)] were added to each cell culture flask and cells were incubated at 37°C and 5% CO_2_. Three to four days later, supernatants were collected and centrifuged at 2575 × *g* for 10 min at 4°C. The supernatant was transferred into high speed centrifugation tubes and centrifuged at 39742 × *g* for 2 h at 4°C. The virus pellet, which was used for further isolation of L- and H-particles, was overlaid with 150 μL of MNT buffer (virus stock for infection experiments; 30 mM MES, 100 mM NaCl, 20 mM Tris) or 150 μL of DMEM without phenol red (high glucose, particle isolation) and stored overnight at 4°C. Virus pellets were resuspended, aliquoted in cryo-vials and stored at −80°C. Virus titration was performed as described elsewhere (Grosche et al., 2019). The BHK21 cell line is one of the cell lines generally used for HSV-1 amplification generating both H- and L-particles. In contrast, mDCs established a mechanism to counteract HSV-1 propagation and only releasing L-particles (Turan et al., 2019).

### Isolation of HSV-1-Derived Particles

For particles derived from HSV-1-infected BHK21 cells, 15 T_175_ cell culture flasks of 90% confluent BHK21 cells were inoculated and supernatants were harvested to pellet the virus as described above in “Cells, virus strain and amplification.” For the generation of L-particles derived from HSV-1-infected mDCs, 50 × 10^6^ to 130 × 10^6^ mDCs were infected with HSV-1 using an MOI of 1 in a total volume of 15 to 25 mL infection medium. After 1 h of incubation at 37°C and gentle shaking, cells were centrifuged at 303 × *g* for 5 min and transferred into DC medium adjusting a final cell concentration of 1.25 × 10^6^ mDCs/mL. At 20 hpi, supernatants of HSV-1-infected mDCs were harvested and virus particles were pelleted as described in “Cells, virus strains and amplification.” Since the lifetime of mDCs is limited and the replication cycle is abortive in mDCs, compared to the complete replication cycle in BHK21 cells, the incubation time of mDCs and BHK21 cells is different. H- and/or L-particles were isolated via a first centrifugation of supernatants from HSV-1-infected cells (centrifuged at 39742 × *g* as described above) according to a previously published protocol (Szilágyi and Cunningham, 1991). In brief, virus suspension was loaded onto a gradient of 5–20% Ficoll PM 400 (Sigma-Aldrich, Deisenhofen, Germany) and centrifuged at 26,000 × *g* for 2 h at 4°C. Subsequently, H- particles and L-particles were collected by punctuation with a needle. Finally, isolated H- and L-particles-containing suspensions were transferred into centrifugation tubes (Beckman Coulter, Brea, United States), filled up with 30 mL DMEM without phenol red and samples were centrifuged at 80,000 × *g* for 2 h at 4°C. The pellets were resuspended in an appropriate amount of DMEM without phenol red according to the virus pellet size and stored at −80°C until further usage. L-particles were UV-irradiated three times applying 0.12 J/cm^2^ in a Vilber Luormat (Biometra, Göttingen, Germany), in order to inactivate contaminating H-particles.

### Electron Microscopy

Electron microscopy was performed as previously described (Heilingloh et al., 2015). Briefly, in a first step, dialysis of H- and L-particle preparations against 20 mM HEPES was performed in a SnakeSkin pleated dialysis tube (10000-moleculare-weight cutoff, Thermo Scientific, Rockford, IL, United States) overnight at 4°C. Afterward, particles were seeded on carbon-coated 400-square-mesh copper grids (Electron Microscopy Sciences, Hatfield, PA, United States) for 20 min at RT and fixed with 2% glutaraldehyde. Finally, particles were stained with 1% uranyl acetate (diluted in 50% ethanol) for 10 min and subsequently with lead citrate for 5 min. For data analysis a transmission electron microscope (Leo 912; Zeiss, Oberkochen, Germany) was used.

### Preparation of Particle Lysates and Immunoblotting

An aliquot of isolated H- and L-particle solutions was mixed with 4× Roti-Load 1 (Carl Roth GmbH, Germany; final concentration: 1×) and denaturated at 95°C for 10 min. Protein samples were loaded onto 10% SDS polyacrylamide gels (SDS-PAA) and separated using SDS-PAGE. Afterward, proteins were transferred onto a nitrocellulose membrane by wet transfer or SDS-gel was incubated with coomassie staining solution (0.05% Coomassie Brilliant Blue R-250 (BioRad 161-0400), 20% isopropanol, 10% acetic acid) at 50°C for 10 min. Subsequently, gels were washed once in 10% acetic acid at RT for 1h and further destained in ddH_2_O overnight at RT. For L-particles derived from mDCs, SDS-gel was stained using the SilverQuest staining Kit (Invitrogen, California, United States) according to manufacturing instructions. In case of Western blotting, the membrane was blocked in 1× Roti-block (Carl Roth GmbH, Germany) for 1 h, and incubated with primary antibodies overnight at 4°C. The antibodies were detected via Image Quant and ECL using Amersham ECL Prime Western blotting detection reagent (GE Healthcare, Solingen, Germany) after the membrane was incubated with a species-specific HRP-conjugated secondary antibody. All used antibodies are listed next: ICP5 antibody (Santa cruz, sc-56989, clone 3B6), gB antibody (Santa cruz, sc-56987, clone 10B7), ICP4 antibody (Santa cruz, sc-56986, clone 10F1), ICP0 antibody (Santa cruz, sc-53070, clone 11060), gD antibody (Santa cruz, sc-21719, clone DL6), ICP8 antibody (Santa cruz, sc-53329, clone 10A3) and UL42 antibody (Santa cruz, sc-53331, clone 13C9) (all primary antibodies were diluted 1:1,000), polyclonal anti-mouse-IgG HRP-linked (Cell signaling, 1:2,500).

### Mass Spectrometry Analyses

For mass spectrometric analyses, we used MS1-based label-free quantification. This method is based on the quantification of peptide signals at the MS1 level after tryptic digestion (Pappireddi et al., 2019). The total peak areas of the peptide are integrated over the time. For subsequent relative quantification, the intensities (“sum of the ion peak intensities of unique peptides”) of each peptide in one experiment is compared to the respective signals in other experiments (Bantscheff et al., 2007). The unique peptides in our analysis are those ones which are used for the MS1-based label-free quantification between the samples.

Samples were prepared as follows: L- and H-particle preparations from HSV-1-infected BHK21 cells and mDCs were lysed in 4× Roti-Load 1 (Carl Roth GmbH, Germany; final concentration: 1×) and subsequently denatured at 95°C for 10 min. Samples were processed for MS1-based label-free quantification by a modified filter-aided sample preparation method (Wiśniewski et al., 2009; Lamm et al., 2017). The 10 kD cutoff filter (Microcon YM-10, Vivacon 500; Sartorius) was washed once with 8 M Urea supplemented with 50 mM Tris (pH 8.0; washing buffer). After each loading or washing step, the filter was centrifuged at 12.000 × *g* until dryness. Approximately 3.5 μg (mDC particles) or 20 μg (BHK21 particles) of sample preparation was mixed with 200 μL washing buffer and a maximum of 300 μL of the sample solution was loaded onto the filter unit. If the sample volume exceeded the maximal volume, sample loading was repeated until the entire sample was loaded. The filter unit was washed three times with washing buffer. Subsequently, samples were reduced using 25 mM DTT in washing buffer at 37°C for 30 min at 600 rpm. Afterward, samples were alkylated with 25 mM chloroacetamide present in the washing buffer at RT for 30 min in the dark. Filter units were washed with washing buffer until foam formation in the flow through disappeared. Protein-loaded filter units were washed with 6 M Urea supplemented with 50 mM Tris (buffer B) and afterward incubated with buffer B supplemented with 50 μL of 0.5 μg Lys-C (Wako Chemicals) at 37°C for 3h at 600 rpm. Subsequently, a final concentration of 1 M urea was adjusted using 50 mM Tris buffer (= dilution buffer) and protein samples were trypsinized using 1 μg trypsin at 37°C and 600 rpm overnight. The filter units were washed with 100 μL washing buffer and the flow through was collected in low binding tubes after centrifugation. The peptides were acidified up to a final concentration of 0.5% TFA. Subsequently, peptide samples were desalted using C18 membrane stage tips, which were activated using 100% acetonitrile (ACN). After a 2 min centrifugation step at 800 × *g*, peptide samples were loaded onto the columns. The columns were washed with 1% ACN supplemented with 0.1% TFA and a defined amount of peptides was eluted into fresh 1.5 mL low binding tubes, using 50% ACN containing 0.1% TFA. Afterward, peptides were vacuum concentrated and resuspended in 1% ACN supplemented with 0.1% TFA. All peptide samples were separated by reverse phase chromatography with a linear increase of acetonitrile on a nano flow Ultimated 3000 HPLC (Dionex) with a flow rate of 200 nL/min. Separated peptides were ionized by an EASY-Spray ion source (Thermo Fisher Scientific) with 2.0 kV and 275°C of the transfer capillary. All samples were analyzed by an Orbitrap Fusion tribrid (Thermo Scientific) working in a positive polarity mode. Detailed mass spectrometry scan settings were previously described (Kraner et al., 2017). Raw data were analyzed using PEAKS Studio 8.5 [Bioinformatics Solutions, Waterloo, Ontario, Canada; (Zhang et al., 2012)] against a combined host-viral database using the HHV1 uniprot.org database [downloaded in November 2018 (BHK21 cell-derived particles) and July 2019 (mDC-derived particles)], *Homo sapien*s uniprot.org database (downloaded in July 2019, mDC-derived particles) or *Mesocricetus auratus* uniprot.org database (downloaded in May 2020, BHK21 cell-derived particles). Oxidation of methionine and carbamidomethylation of cysteines were set as dynamic and static modifications, respectively. Proteins included into data interpretation must exhibit a false discovery rate of <1%. For evaluation of the data we normalized the intensity of each protein to the mean of all glycoproteins detected in three mDC or four BHK21 particle preparations. The analyzed data sets represent three or four replicates for mDC- or BHK21 cell-derived HSV-1 particles, respectively.

### Infection of iDCs and Flow Cytometric Analyses of the Maturation Phenotype

To analyze DC activation phenotype prior and post incubation with HSV-1 virions or HSV-1-derived L-particles, 0.6 × 10^6^ cells were mock- or HSV-1-infected (MOI of 2) or treated with UV-inactivated HSV-1 (viral material corresponding to MOI of 20, 8 × 0.12 J/cm^2^) or HSV-1 L-particles (viral material corresponding to high MOI, 3 × 0.12 J/cm^2^). The infection was performed in 300 μL infection medium supplemented with the defined amount of HSV-1-derived virions or L-particles. At 1 hpi, iDCs including the infection medium were transferred directly into DC medium containing the maturation cocktail as described in “Generation of dendritic cells” in a final concentration of 1 × 10^6^ cells/mL. Cells were harvested 24 hpi and prepared for flow cytometric analyses. In brief, cells were washed once with FACS buffer (PBS containing 2% FCS) and incubated with LIVE/DEAD Fixable Violet dead cell stain (Life Technologies, Carlsbad, CA, United States) and specific antibodies against mouse anti-human CD11c (BD Bioscience, PE-Cy5, clone: B-ly6), mouse anti-human CD83 (Invitrogen, APC, clone: HB15e), mouse anti-human CD80 (BD Pharmingen, PE, clone: L307.4), rat anti-human CCR7 (CD197) (BD Pharming, PE-Cy7, clone: 3D12), anti-human HLA-DR (Bio Legend, APC/Cy7, clone: L243) at 4°C for 30 min in the dark. Finally, cells were washed two times with FACS buffer, fixed with 2% PFA in FACS buffer and measured using FACS Canto II flow cytometer (BD) and analyzed with FCS express five flow research edition software.

### Mixed Lymphocyte Reaction (MLR)

Mature DCs were infected with purified H-particles (MOI of 2), incubated with UV–inactivated H-particles (8 × 0.12 J/cm^2^, viral material corresponding to MOI of 20) or purified L-particles (viral material corresponding to high MOI, 3 × 0.12 J/cm^2^) or left untreated (mock control). At 8 hpi, cells were harvested and titrated numbers of mDCs were cocultured with 0.4 × 10^6^ allogeneic T cells derived from the non-adherent fraction of PMBCs (Pfeiffer et al., 2013). After additional 72 h of incubation in a 96-well flat bottom plate, cocultures were pulsed with 1 μCi/well [3H]-thymidine (PerkinElmer) for 16 h before they were harvested onto glassfiber filtermates using an ICH-110 harvester (Inotech). Counts per minutes were measured in a 1450 microplate counter (Wallac). For the evaluation of the MLR, the mean of the duplicates for each condition was calculated.

### Statistical Analyses

Results of mass spectrometric analyses are displayed as individual intensity per replicate or mean intensity of all replicates as stated in the respective figure legend. Data regarding DC maturation phenotype or MLR are shown as median or counts per minutes ± standard error of the mean (SEM) as indicated. For the determination of the significance, data were analyzed using one-way analysis of variance (ANOVA) and Bonferroni’s multiple-comparison *post hoc* test. Significance was accepted for p-values less than 0.05. ^****^indicates *p* ≤ 0.0001; ^∗∗∗^*p* ≤ 0.001; ^∗∗^*p* ≤ 0.01; ^∗^*p* ≤ 0.05; and ns, not significant.

### Approvals and Legal Requirements

The local ethics committee has given the permission to generate monocyte-derived DCs from leukapheresis products of healthy donors (reference number: 184_16Bc). This study was carried out in accordance with the recommendations of the ethics committee of the “Friedrich-Alexander-Universität Erlangen-Nürnberg,” with written informed consent from all subjects. All subjects gave written informed consent in accordance with the Declaration of Helsinki.

## Results

### Distinct Viral Protein Distribution Among BHK21 Cell- and mDC-Derived HSV-1 L-Particles

**FIGURE 1:**
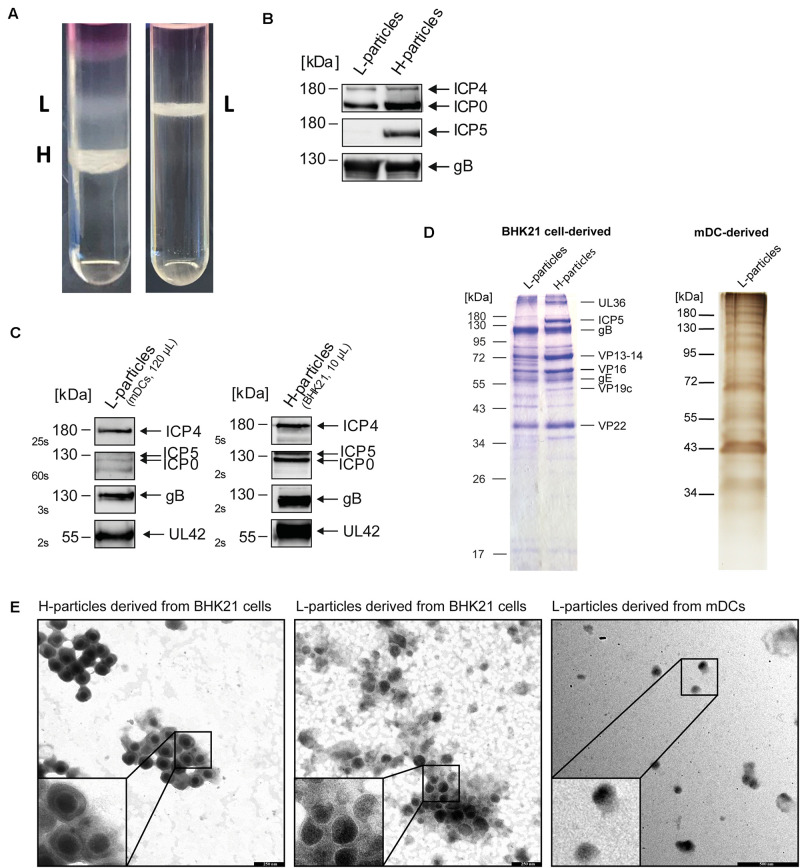
Purification of L- and H-particles derived from HSV-1-infected BHK21 cells or mDCs. L-particles and H-particles were purified from supernatants of HSV-1-infected BHK21 cells and mDCs four days post infection and 20 hpi, respectively. **(A)** Representative Ficoll gradients from HSV-1-infected BHK21 cells (left panel) and mDCs (right panel) are shown. **(B)** Western blot analyses of L- and H-particle protein lysates derived from BHK21 cells. A total protein amount of approximately 4 μg was loaded onto a 10% SDS gel. **(C)** Western blot analyses of L-particle protein lysates derived from HSV-1-infected mDCs (left panel) in comparison to BHK21 cell-derived H-particles (right panel). **(B,C)** Purity of particle preparations was verified by detection of ICP5, ICP4, ICP0, and gB using specific antibodies. Exposure time for detection is depicted as seconds (s). **(D)** Coomassie blue staining of H- and L-particle samples isolated from HSV-1-infected BHK21 cells loaded onto a 10% SDS-PAA gel (left). Silver staining of L-particles derived from HSV-1-infected mDCs loaded onto a 10% SDS-PAA gel (right). **(E)** Electron microscopic analyses of HSV-1 particles derived from either BHK21 cells or mDCs. HSV-1 particles were adhered to carbon-coated grids and stained using 1% uranyl acetate and lead citrate. Bars, 250 nm (particles derived from BHK21 cells) and 500 nm (L-particles derived from mDCs).

In a comparative analysis, the protein composition of L- and H-particles from HSV-1-infected BHK21 cells and L-particles from HSV-1-infected mDCs was examined using MS1-based label-free quantification. For this, BHK21 cells and mDCs were infected with HSV-1 and supernatants of infected cells were harvested after four days and 20 h post infection (hpi), respectively. The difference in the incubation time is due to the limited life time of DCs and the abortive replication cycle in mDCs compared to complete replication in BHK21 cells. Subsequently, L- and H-particles were isolated using a Ficoll gradient followed by ultracentrifugation. As expected, HSV-1-infected BHK21 cells release both H- and L-particles (Figure 1A, left panel). As recently reported, the release of H-particles by HSV-1-infected mDCs is limited due to the block of nuclear egress by the inhibition of autophagic flux upon DC maturation (Turan et al., 2019). Figure 1A (right panel) confirms this observation, since the supernatants of HSV-1-infected mDCs almost exclusively contained L-particles and only barely detectable amounts of H-particles. Prior to mass spectrometric analyses, a potential H-particle contamination in the L-particle preparations was verified by the absence of major capsid protein ICP5 in L-particle protein lysates (Figure 1B). Moreover, the detection of ICP4, ICP0, gB, and UL42 confirmed the successful isolation of sufficient amounts of either L-particles (mDCs) or H-particles (BHK21 cells; Figure 1C). Apart from Western blot detection, the overall protein distribution in L- and H-particles preparations from HSV-1-infected BHK21 cells and mDCs was analyzed via coomassie blue and silver staining of the SDS-PAA gels, respectively (Figure 1D). Based on this, a distinct viral protein pattern of both particle variants as well as the exclusive presence of ICP5 and VP19c in H-particles was observed. Regarding the molecular weight of selected viral proteins, the most likely candidates are indicated and revealed the lower abundance of VP13-14 (UL47) in L-particles compared to H-particles (Figure 1D). To verify the purity of L- and H-particle fractions, we analyzed the preparations via electron microscopy (Figure 1E). The fraction of the BHK21-derived H-particles showed the characteristic nucleocapsid (black circles within virions) surrounded by the viral envelope. In contrast, the HSV-1 L-particle fractions derived from BHK21 cells and mDCs were more heterogeneous in size, compared to full virions, and are lacking the viral capsid. The here presented data clearly show that we used pure particle preparations.

**FIGURE 2:**
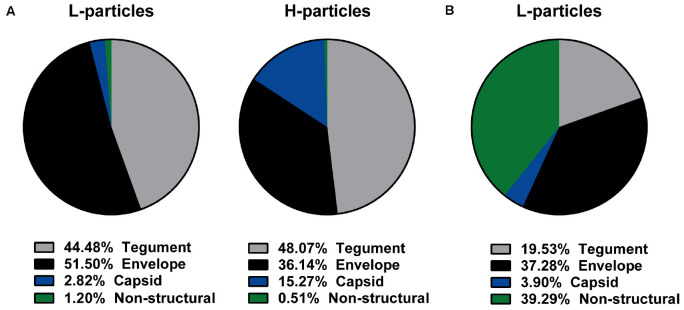
Viral protein distribution of HSV-1-derived particles isolated from infected BHK21 cells or mDCs. **(A,B)** L-particles and H-particles were purified from supernatants of HSV–1-infected BHK21 cells **(A)** and mDCs **(B)** four days post infection and 20 hpi, respectively. Protein distribution of L- and H-particles was analyzed by mass spectrometry. The total intensity of viral proteins was set to 100% and relative values (normalized to the mean of all present glycoproteins) for H- and L-particle samples are shown regarding their predicted protein localization. The data are based on four BHK21cell- or three mDC-derived particle preparations.

Subsequently, MS1-based label-free quantification was performed to decipher these differences in the protein pattern. We used four independent L- and H-particle preparations from HSV-1-infected BHK21 cells and three L-particle preparations isolated from HSV-1-infected mDCs. Figure 2 shows the schematic distribution of all detected viral proteins regarding their predicted localization in HSV-1 particles from BHK21 cells (Figure 2A) or mDCs (Figure 2B). H- and L-particles derived from HSV-1-infected BHK21 cells comprised 63 viral proteins, while L-particle samples from mDCs contained 41 viral proteins. The mean intensity of each viral protein of all samples was calculated and all viral proteins were sorted regarding their predicted localization. Finally, the total intensities of tegument, envelope, capsid and non-structural proteins were set relative to the total intensity of all detected proteins.

Since L-particles are characterized by the lack of the viral capsid, only ∼3% (BHK21 cells) to ∼4% (mDCs) of the overall protein quantity were capsid-associated proteins, whereas this percentage was five-fold higher in H-particle preparations from BHK21 cells (Figures 2A,B). Moreover, the marginal incorporation of capsid proteins in L-particles derived from HSV-1-infected BHK21 cells seemed to be substituted by envelope proteins, since half of all viral L-particle proteins (51.50%), but only one third (36.14%) of all viral H-particle proteins, were found within envelope proteins. In contrast, the percentage of tegument proteins was unaltered between both particle types isolated from BHK21 cells (44–48%). Thus, our mass spectrometric data reveal a shift regarding the percentage of the viral protein distribution in L-particle compared to H-particle preparations derived from HSV-1-infected BHK21 cells, i.e., from capsid toward envelope proteins. L-particles derived from HSV-1-infected mDCs showed a different overall protein distribution, characterized by the extremely high abundance of viral proteins annotated as non-structural proteins, compared to BHK21 cell-derived L-particles (Figures 2A,B). Approximately three quarters of all detected viral proteins in L-particles from HSV-1-infected mDCs were associated with the envelope (37.28%) or were categorized as non-structural proteins (39.29%), whereas only ∼20% of the viral proteins were localized in the tegument. Taken these results, L-particles derived from HSV-1-infected mDCs seem to abundantly incorporate distinct viral proteins, i.e., annotated as non-structural proteins, compared to those derived from BHK21 cells.

### Highly Sensitive Mass Spectrometric Analysis Reveals 63 and 41 Viral Proteins in Viral Particle Preparations of HSV-1-Infected BHK21 Cells and mDCs, Respectively

Apart from comparing the overall protein distribution in L- versus H-particles derived from different HSV-1-infected cell types, changes in the viral protein pattern were analyzed for each identified viral protein individually. The following tables summarize all detected viral proteins in H- and L-particle preparations according to their localization in either of the particle types (Tables 1, 2). Moreover, the detection coverage as well as the number of unique peptides is depicted. In the last decades, the composition of L-particles has been barely investigated, recently only one research group analyzed viral proteins incorporated into L-particles and H-particles in a mass spectrometry-based attempt (Russell et al., 2018). In particular, Russell et al. (2018). identified 51 and 42 viral proteins in preparations of H- and L-particles, respectively, derived from HSV-1- strain Sc16-infected HaCaT cells. Our mass spectrometric data extend this previous report based on the identification of 63 viral proteins in both L- and H-particle samples from HSV-1 strain 17-infected BHK21 cells. Among viral proteins in particles derived from BHK21 cells we identified 24 tegument proteins, 16 proteins belonging to the envelope and eight were annotated as capsid proteins. The remaining 15 viral proteins were designated as non-structural proteins. Furthermore, we affirmed the incorporation of all recently discovered tegument and glycoproteins which have been identified in previous publications (Loret et al., 2008; Loret and Lippé, 2012; Russell et al., 2018). Concerning ICP34.5, which represents one notable exception, our HSV-1 particle preparations are in line with those reported by Russell et al. (2018), also documenting the absence of this viral protein, whereas previously published mass spectrometric analyses reported the presumed presence of this viral protein based on the detection of a single unique peptide (Loret et al., 2008). Beyond this, our data confirmed the presence of UL20, UL34 and the glycoproteins gK and gN and we successfully identified all five IE proteins, i.e., ICP0, ICP4, ICP22, ICP27, ICP47 and DNA-associated proteins, such as ICP8, UL5, UL30, UL42, and UL52.

**TABLE 1:** Mass spectrometric identification of viral proteins in L-particles derived from HSV-1-infected mDCs.

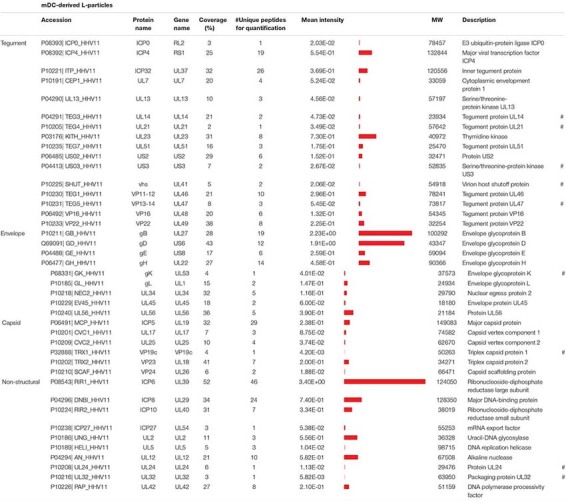

*L-particles were purified from supernatants of HSV−1-infected mDCs cells using a Ficoll gradient. Subsequently, samples were prepared for mass spectrometric analyses. Proteins are summarized regarding their predicted localization in the viral particle in numerical order and mean intensities of all three replicates are normalized to mean of all glycoproteins detected in at least two out of three samples. “MW” means molecular weight. Proteins detected in two out of three samples are marked with “#.”*

**TABLE 2:** Mass spectrometric identification of viral proteins in H-particles and L-particles derived from HSV-1-infected BHK21 cells.

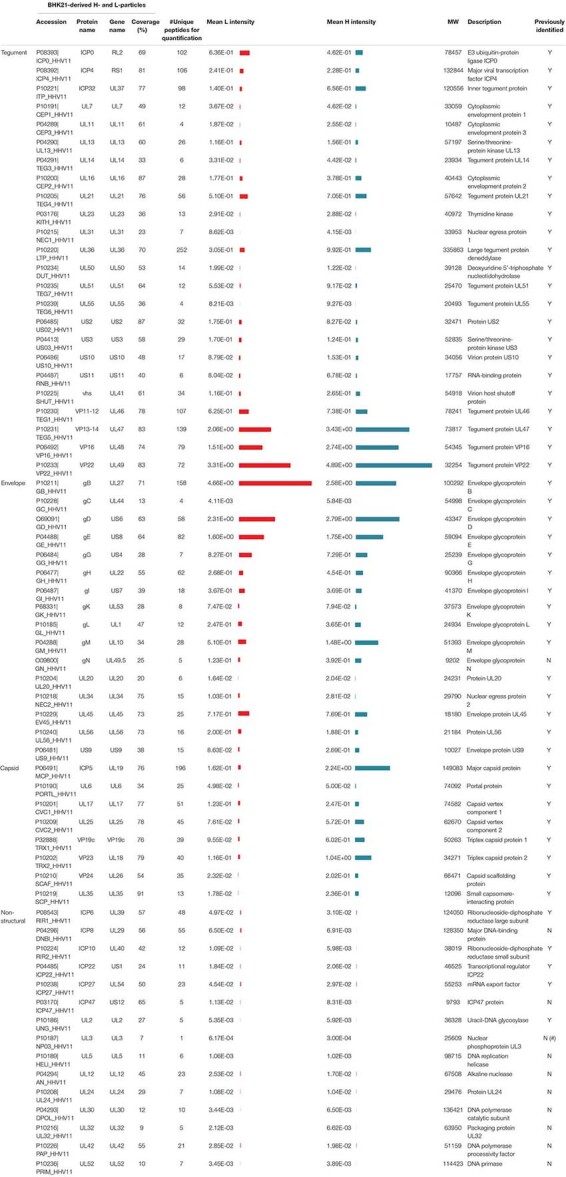

*L-particles and H-particles were purified from supernatants of HSV−1-infected BHK21 cells using a Ficoll gradient. Subsequently, samples were prepared for mass spectrometric analyses. Proteins are summarized regarding their predicted localization in the viral particle in numerical order. The shown intensity represents the mean intensity of all four samples normalized to the mean of all detected glycoproteins. Previously identified proteins are indicated with yes (“Y”) or no (“N”) (Loret et al., 2008; Russell et al., 2018). “MW” means molecular weight. Proteins detected in two out of three samples are marked with “#.”*

More importantly, 41 viral proteins were also detected in L-particles released by HSV-1-infected mDCs. We identified 16 proteins annotated as tegument proteins, nine as envelope proteins, six as capsid proteins and 10 viral proteins as non-structural proteins. Our data of MS1-based label-free quantification further demonstrate that L-particles released by HSV-1-infected mDCs contained only three out of five viral IE proteins (ICP0, ICP4, and ICP27) and high amounts of the glycoproteins gB and gD. As already observed in the overall protein distribution (Figure 2), the intensity of non-structural proteins is notably higher in mDC-derived, compared to BHK21 cell-derived, HSV-1 L-particles (Tables 1, 2).

### L-Particles From HSV-1-Infected BHK21 Cells Contain H-Particle-Associated Proteins at Different Quantities

**FIGURE 3:**
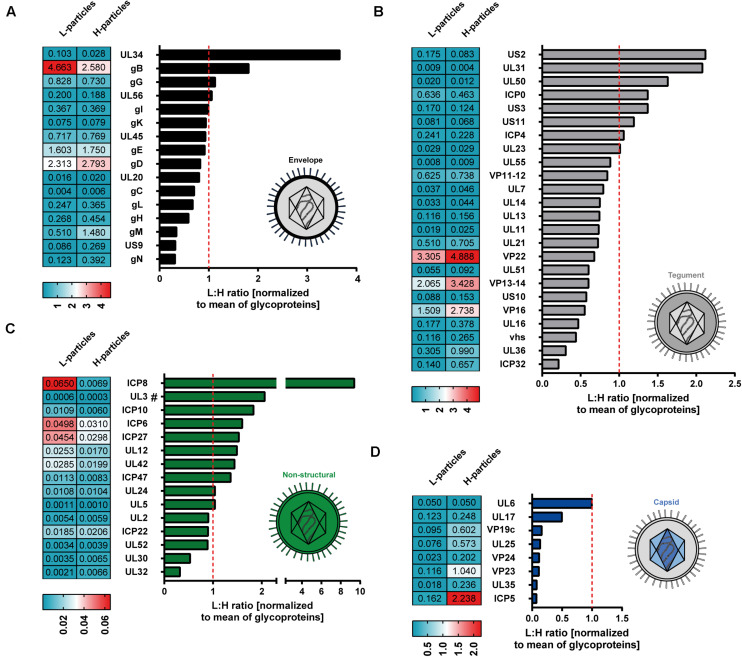
Mass spectrometry-based comparative analysis of HSV-1-derived L-particles versus H-particles from infected BHK21 cells. HSV–1-derived H- and L-particles from supernatants of infected BHK21 cells were isolated for subsequent mass spectrometric analysis. The results of all four replicates are shown as relative ratio of L- versus H-particles using the respective protein intensity normalized to the mean intensity of all detected glycoproteins. Proteins are grouped according to their annotation as envelope **(A)**, tegument **(B)**, non-structural **(C)** or capsid proteins **(D)** and ordered regarding their ratio. The dashed red line represents a ratio of 1. UL3 was detected in two out of three samples and is marked with “#.” The values in the heat maps represent the average intensity of each detected protein among different samples, normalized to the mean of all detected glycoproteins, in the respective samples. Normalized values were separately calculated for L- and H-particle samples.

Decoding the protein pattern of L-particles is essential to get deeper insights into the biological role of these non-infectious particles during an HSV-1 infection. Our mass spectrometry-based data revealed 63 viral proteins to be incorporated into HSV-1-derived H- and L-particles produced by infected BHK21 cells. The intensity of these detected viral proteins was normalized to the respective mean intensity of all glycoproteins detected in the respective sample preparation. Subsequently, the ratio of L- versus H-particles was determined for each normalized value (Figure 3). However, a higher ratio of a protein in L-particles does not equate a higher abundance in absolute amounts.

The envelope represents the glycoprotein-rich outer membrane of herpesvirus particles. As shown in Figure 2A, L-particles contained higher levels of specific envelope proteins than H-particles (Figure 3A). Exclusively two proteins of this group, i.e., UL34 and gB, were significantly overrepresented in L-particles. In sharp contrast, gC, gL, gH, gM, US9, and gN were detected at lower amounts in L-particles compared to H-particles, however, gL, gH, and gM were still present in L-particles (see values beside Figure 3A). Interestingly, two third of tegument proteins were underrepresented in L-particles, compared to H-particles (Figure 3B, ratio <1). In contrast, our data revealed that the tegument proteins US2, UL31, UL50, ICP0, and US3 were more abundant in L-particles compared to their infectious counterparts (∼1.4 to 2.2-fold). Two proteins, namely ICP4 and the viral thymidine kinase (UL23), were almost similarly integrated into both particle types (ratio ∼1). As expected, the here presented data of MS1-based label-free quantification confirmed that capsid related proteins were significantly underrepresented in L-particles from HSV-1-infected BHK21 cells compared to H-particle preparations, indicating a high purity of these L-particle preparations (Figure 3C). One notable exception was UL6, the portal forming protein for entry of viral DNA into the capsid, which was found to be equally distributed among L- and H-particles from BHK21 cells (Newcomb et al., 2001; Trus et al., 2004). Moreover, our data clearly showed that the capsid proteins UL17 and UL25, promoting the insertion and retention of viral DNA, were present in H-particle samples as already described to be incorporated into HSV-1 virions (McNab et al., 1998; Salmon et al., 1998; Toropova et al., 2011). Furthermore, regarding the L-particle composition, our mass spectrometric data are in line with previous reports showing that the two capsid proteins UL17 and UL6 are present in L-particles (Thurlow et al., 2005). Our results show that the major capsid protein, ICP5, was more abundant in H-particles compared to L-particles, resulting in the lowest L- to H-particle ratio regarding capsid proteins. However, we cannot exclude the possibility of small H-particle contaminations in our L-particle preparations. The remaining non-structural proteins mainly include proteins that were overrepresented in L-particles. For example, ICP8, the major DNA binding protein, is one of the strongest differentially incorporated protein in L-particles compared to H-particles, revealing a 10-fold higher abundance in L-particles (Figure 3D). However, it has to be pointed out that the overall intensity of these non-structural proteins is very low in general and thus the comparison of non-structural proteins in BHK21 cell-derived HSV-1 particles should be treated with caution. In conclusion, our data demonstrate the presence of 63 viral proteins in H- and L-particles which are integrated to distinct extents into either of both particle types derived from infected BHK21 cells.

### HSV-1 L-Particles Derived From Infected mDCs Contain High Amounts of gB, gD, ICP6, ICP4, and UL23

**FIGURE 4:**
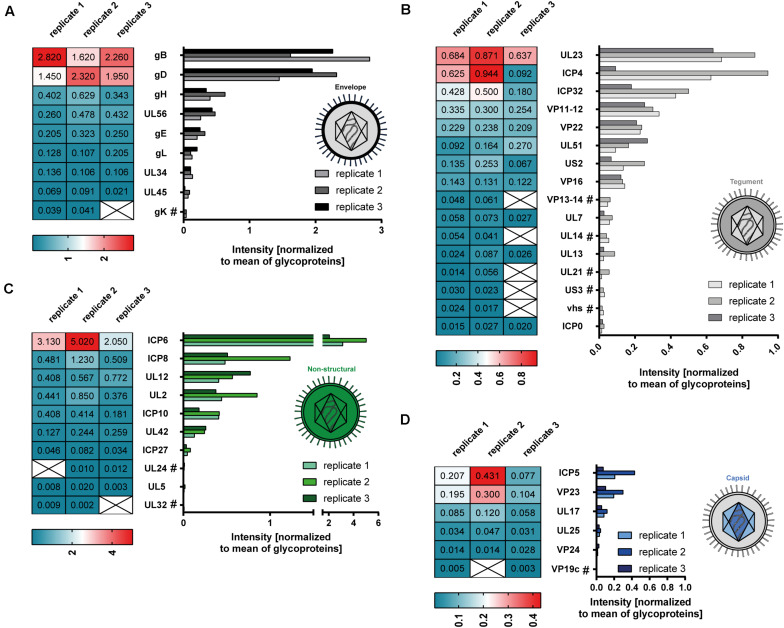
Mass spectrometry-based analysis of HSV-1-derived L-particles from mDCs. HSV–1-derived L-particles were isolated from supernatants of infected mDCs and used for subsequent mass spectrometric analysis. The results of all three replicates are shown as individual intensities normalized to the mean intensity of all glycoproteins in the respective sample (heat map, graph). Proteins are grouped according to their annotation as envelope **(A)**, tegument **(B)**, non-structural **(C)** or capsid proteins **(D)**. Proteins detected in two out of three replicates are marked with “#.”

Our mass spectrometry-based data revealed 41 viral proteins to be incorporated into L-particles produced by HSV-1-infected mDCs. Figure 4 illustrates the individual intensities of all detected viral proteins, normalized to the mean intensity of all glycoproteins, in the three mDC L-particle replicates, depicted as bar graphs or heat maps. The most abundant viral proteins were associated with the envelope or non-structural proteins. As already shown by our initial Western blot analyses, L-particles from mDCs contained high amounts of ICP4, gB and UL42 (Figure 1C). These observations, including the low presence of ICP0 in L-particles and the high abundancy of gB and gD as major envelope proteins, were confirmed by our mass spectrometric results (Figures 4A–C). Again, capsid-associated viral proteins were barely detectable in our L-particle samples from HSV-1-infected mDCs, which confirms the lack of viral capsids in these preparations (Figure 4D). Since HSV-1-infected mDCs predominantly produce L-particles (Figure 1A), we exclude the possibility of significant H-particle contaminations in our mDCs preparations. Regarding proteins predicted within the tegument, we detected the UL23 as the most abundant protein, which is responsible for the phosphorylation of thymidine and thus the production of nucleotides for viral DNA synthesis (Chen et al., 1998; Pilger et al., 1999; Xie et al., 2019). Furthermore, we detected the viral protein ICP32, which is known as larger inner tegument protein, responsible for cytoplasmic secondary envelopment (Owen et al., 2015). Also the functionally important viral protein VP16, initializing the viral IE gene expression cascade, was identified in these L-particle preparations (Figure 4B; Triezenberg, 1995). Surprisingly, apart from the high abundant HSV-1-encoded proteins gB and gD in L-particles derived from HSV-1-infected mDCs, we also detected high amounts of ICP6, which is categorized in non-structural proteins. In summary, L-particles produced by HSV-1-infected mDCs are highly coated by the two glycoproteins gB and gD and contain three of the five known IE proteins (ICP0, ICP4, ICP27).

### HSV-1-Derived L-Particles Possess a Cell Type-Dependent Unique Protein Signature

**FIGURE 5:**
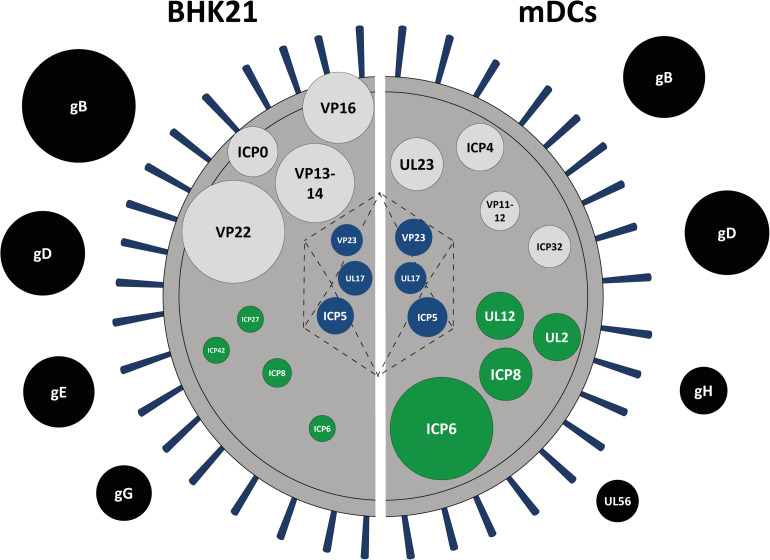
Schematic protein signature of HSV-1 L-particles derived from HSV-1-infected BHK21 cells and mDCs. Graphical comparison of the viral protein signature of HSV-1 L-particles derived from infected BHK21 cells (left part) and mDCs (right part) based on the mean intensity of all samples (BHK21: four samples, mDCs: three samples) as shown in Tables 1, 2. The top abundant hits are shown as circles, each representing a single viral protein. The diameter was adapted according to the relative signal intensities of the depicted viral proteins. The proteins are categorized regarding their predicted localization within the virion, i.e., tegument (black), envelope (gray), capsid (blue), or non-structural proteins (green).

For better illustration and comparison between L-particles released by HSV-1-infected BHK21 cells and mDCs, the top three to four abundant proteins for each protein group are schematically depicted in Figure 5. However, L-particles are derived from two different cell types and measurements and thus the detection of different peptides is not linear. Regarding HSV-1 L-particles derived from either of the two cell types, we revealed that both preparations shared some characteristics, i.e., the presence of particular glycoproteins and non-structural proteins. Especially the high abundancy of gB and gD were comparable in both particle preparations from BHK21 cells and mDCs (Figures 3A, 4A, 5). Even though the intensity of the non-structural proteins strongly differed between L-particles from BHK21 cells and mDCs, the major hits, e.g., ICP6 and ICP8, were identical (Figure 5, green circles). However, the distribution among tegument and capsid proteins was inconsistent between L-particles from BHK21 cells and mDCs. In particular, L-particles from HSV-1-infected BHK21 cells contained high amounts of VP22, VP13-14, ICP0, and VP16 protein, while the major hits in mDC-derived HSV-1 L-particles were UL23, ICP4, VP11-12 and ICP32. Although the capsid and thus the viral genome is not present in L-particles, the major capsid protein ICP5 could, however, in some degree be detected in L-particles derived from HSV-1-infected BHK21 cells as well as mDCs (Figures 3D, 4D, 5). Notwithstanding, it is very likely that capsid proteins are incorporated into L-particles, whereas progeny capsids are absent in this non-infectious HSV-1 particle structure. In conclusion, our data indicate that the composition of HSV-1-derived L-particles is reflected by the producing cell type.

### Host Cell Protein Composition in BHK21- and mDC-Derived Particles

**FIGURE 6:**
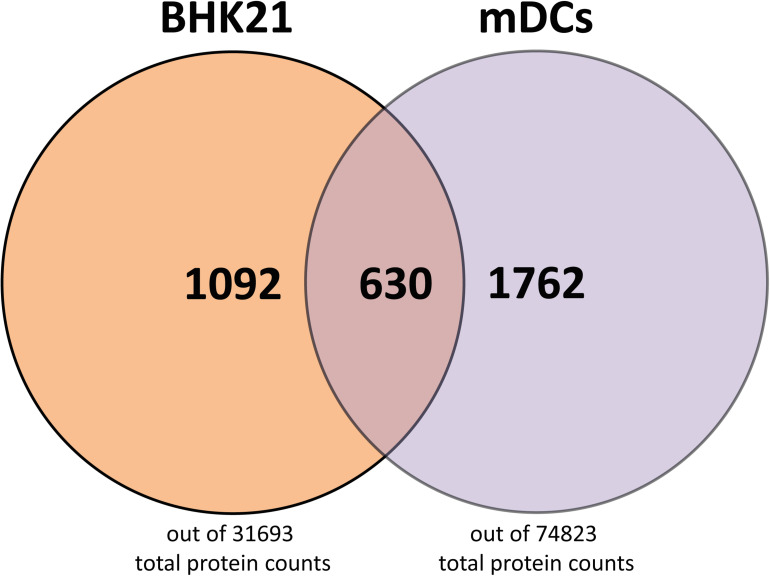
Host cell proteins in L- and H-particles derived from BHK21 cell and L-particles derived from mDCs. A total number of host cell proteins of 1092 were found in H- and L-particles from HSV-1-infected BH21 cells, whereas 1762 host cell proteins were detected in L-particles derived from HSV-1-infected mDCs. 630 host cell proteins were identical in the HSV-1 particles derived from these different cell types. Data is evaluated based on the databases from *Mesocricetus auratus* (BHK21 cells) and *Homo sapiens* (mDCs).

Having analyzed the viral protein composition of BHK21cell- and mDC-derived particles, we next investigated host cell protein incorporation in HSV-1 particles. We detected 1,092 (out of 31,693 total protein count in *Mesocricetus auratus*) and 1,762 (out of 74,823 total protein count in *Homo sapiens*) host cell proteins in BHK21 cell-derived and mDC-derived particles, respectively (Figure 6). The detected host cell proteins in BHK21 cell- and mDC-derived HSV-1 particles, as well as the 630 proteins which are found in the particle types from different cell types, are shown in the supplementary section (Supplementary Tables S1–S3). However, the detection of host cell proteins does not allow the conclusion that they are also incorporated into particles, since we cannot exclude small contaminations of extracellular particles.

### HSV-1-Derived L-Particles Specifically Modulate CD83 Expression During DC Activation and Inhibit DC T Cell Stimulatory Capacity

L-particles derived from both HSV-1 infected BHK21 cells and mDCs contain a plethora of viral proteins (Tables 1, 2). To further analyze the functional role of L-particles during an HSV-1 infection, it was interesting to investigate whether non-infectious particles are able to interfere with DC maturation or with the T cell stimulatory capacity of mDCs.

**FIGURE 7:**
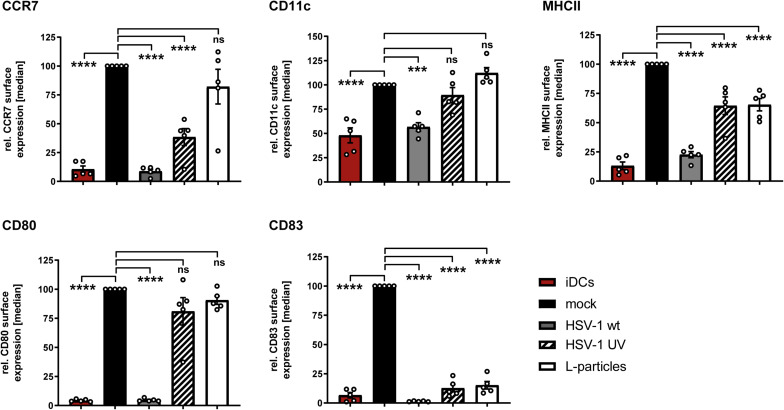
L-particles selectively interfere with CD83 surface expression during DC maturation. Immature DCs (iDCs) were infected with HSV-1 (MOI of 2), treated with UV-inactivated HSV-1 (viral material corresponding to MOI of 20, 8 × 0.12 J/cm^2^) or L-particles (viral material corresponding to high MOI, 3 × 0.12 J/cm^2^) or left untreated. At 1hpi, infected cells were transferred into medium containing a defined maturation cocktail. An uninfected iDC sample served as an input control and was directly stained against CD11c, CD80, CD83, CCR7, and MHC class II (MHCII) for flow cytometric analyses. Cells were harvested 24 hpi, stained with specific antibodies mentioned before and used for flow cytometry. The experiment was performed five times with cells from different healthy donors. Error bars indicate SEM. Significant changes to mock were analyzed using a one-way ANOVA and Bonferroni multiple comparison *post hoc* tests and are indicated by asterisks (***indicates *p* ≤ 0.001 and ****p ≤ 0.0001) and values depicted as “ns” were not significant (*p* < 0.05).

In humans, the uptake of foreign antigens by iDCs in the periphery causes fundamental changes in their phenotype as well as their migratory behavior (Lanzavecchia and Sallusto, 2001; Alvarez et al., 2008). This process is known as DC maturation, which is pivotal for the ability to stimulate naive T cells. Previous studies revealed that the infection of iDCs with HSV-1 impedes their maturation into antigen presenting mDCs (Salio et al., 1999; Grosche et al., 2020). To investigate the impact of L-particles on DC maturation, iDCs were treated with L-particles, HSV-1 virions or MNT buffer, serving as a control, followed by the addition of a defined DC maturation cocktail. Since L-particles are lacking the viral genome and are replication incompetent, UV–inactivated virions are used as an additional control to prove the effect of viral DNA on the observed phenotype. The immature phenotype of these DCs is characterized by moderate expression levels of CD11c, low expression of the chemokine receptor CCR7, as well as the costimulatory molecule CD80, the functionally important CD83 protein and MHC class II molecules which are important for peptide-antigen presentation (von Rohrscheidt et al., 2016; Düthorn et al., 2019). Upon treatment of iDCs with the maturation cocktail, mock cells efficiently undergo maturation, which was reflected by the upregulation of all analyzed activation surface molecules (Figure 7). As previously reported, HSV-1 virions block DC maturation and efficiently inhibit the upregulation of CCR7, MHC class II, CD80 and CD83 (Salio et al., 1999; Grosche et al., 2020). Interestingly, our data demonstrate that L-particles derived from BHK21 cells failed to inhibit the phenotypic maturation of iDCs toward mDCs, since we observed elevated surface expression of CCR7, CD80 and CD11c, comparable to mock-treated controls (Figure 7). Also the phenotype of DCs treated with UV-irradiated HSV-1-virions was comparable to the expression pattern of DCs treated with BHK21-derived L-particles, except for the slightly decreased CCR7 expression. In sharp contrast, L-particles were sufficient to completely hamper CD83 upregulation. Whether L-particles actively mediate the downregulation of CD83 or block the upregulation of this important surface molecule during DC maturation has to be clarified in future studies.

**FIGURE 8:**
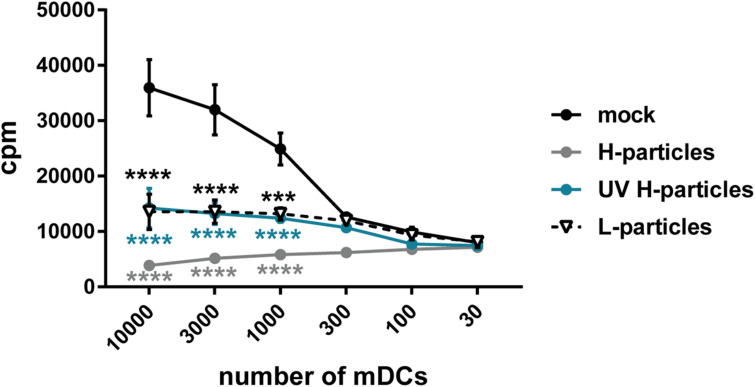
L-particles possess an immunomodulatory effect on mDCs. Mature DCs (mDCs) were infected with purified H-particles (MOI of 2), treated with UV–irradiated H-particles (viral material corresponding to MOI of 20, 8 × 0.12 J/cm^2^) or L-particles (viral material corresponding to high MOI, 3 × 0.12 J/cm^2^) or left untreated (mock). At 8 hpi, cells were cocultured with allogeneic T cells in a mixed lymphocyte reaction (MLR) for additional 72 h. Cells were pulsed with 1 μCi/well [3H]-thymidine (PerkinElmer) for 16 h before harvesting. The experiment was performed three times with mDCs from different healthy donors. Error bars indicate SEM. Statistical analyses was performed relative to mock values and is depicted on the right side. Only the significant changes to mock were analyzed using a one-way ANOVA and Bonferroni multiple comparison *post hoc* tests and only significant changes are indicated by asterisks (*indicates *p* ≤ 0.05, **p ≤ 0.01, ***p ≤ 0.001, and ****p ≤ 0.0001).

Due to the considerable amount of viral proteins and the above described impairment of CD83 upregulation during maturation of iDCs, it is tempting to speculate that L-particles contribute to efficient HSV-1 replication and/or downmodulation of DC-mediated antiviral immune responses in the human host. For human mDCs, it has been shown *in vitro* that infectious virions reduce the T cell stimulatory capacity of these infected mDCs (Kruse et al., 2000). Therefore, it was of high interest to investigate whether also non-infectious L-particles impair the T cell stimulatory capacity of mDCs. Thus, functional mixed lymphocyte reaction (MLR) assay was performed, using mDCs infected for 8 h either (i) with pure HSV-1 virions (H-particles), treated (ii) with UV–irradiated H-particles or (iii) L-particles (all derived from BHK21 cells), or (iv) MNT buffer as mock control. As shown in Figure 8 (black line), mock treated mDC very potently induce T cell proliferation. In sharp contrast, mDCs infected with H-particles were almost completely inhibited in their T cell stimulatory capacity (gray line). Noteworthy, and highly interesting, also mDCs which have been incubated with L-particles derived from BHK21 cells (black dashed line) or UV-inactivated H-particles (blue line) revealed a strongly hampered T cell stimulatory capacity. In conclusion, these functional analyses support our hypothesis that L-particles modulate DC biology and function.

## Discussion

During co-evolution, HSV-1 has evolved several immune evasion mechanisms to subvert the host’s immune response and to particularly interfere with DC biology and function. Recent experiments showed that nuclear lamin proteins are degraded in HSV-1-infected iDCs via hijacking cellular autophagy, which in turn enables the nuclear egress of viral capsids and pilots the release of full mature virions (Turan et al., 2019). Conversely, in HSV-1-infected mDCs, autophagic flux is inhibited via the upregulation of KIF proteins preventing the nuclear egress of viral capsid. As a result, mainly capsidless L-particles and only marginal amounts of mature virions are released into the supernatants of HSV-1-infected mDCs (Figure 1A). However, the production of L-particles is a common process observed among all alpha herpesviruses or during the infection of different cell types (Figure 1A; Dargan and Subak-Sharpe, 1997). Since L-particles derived from HSV-1-infected BHK21 cells and mDCs have been described to affect CD83 surface expression on directly HSV-1-infected and bystander mDCs (Heilingloh et al., 2015), their viral protein composition was analyzed via MS1-based label-free quantification in the present study. In addition, the role of these L-particles in respect to the functional impairment of DCs was studied in more detail.

In general, our mass spectrometric data regarding H-particles derived from BHK21 cell are comparable to the results previously published by other groups (Loret et al., 2008; Russell et al., 2018). Especially the major hits of the capsid (ICP5, VP23), tegument (VP22, UL47, UL36) and envelope proteins (gB, gD, gE) are similar to those provided by Loret et al. (2008). Concerning the abundancy of gB in the viral envelope or U_*S*_11 in the tegument, previous studies revealed a higher tendency in L-particles compared to H-particles (Russell et al., 2018). The envelope protein gC was almost absent in the H- and L-particle preparations derived from HSV-1-infected BHK21 cells as well as in L-particles derived from mDCs (Figures 3A, 4A). As previously published, the virus stock strain 17 syn^+^, which was used in the present study, is heterogenic regarding the gC coding region (Cunha et al., 2015; Jones et al., 2019). This means, that different gC variants are present in the virus stock strain 17 syn^+^. Since some variants miss the transmembrane domain (Jones et al., 2019), this might explain the almost complete absence of gC in our particle preparations. These observations were confirmed by our MS1-based label-free quantification (Figures 3A,B). Taken together, we revealed ∼86% (63 viral proteins) and ∼56% (41 viral proteins) of all annotated viral HSV-1 strain 17 proteins (73 proteins) to be incorporated into HSV-1-derived particles from infected BHK21 cells and mDCs, respectively (UniProt, Reference Proteome, Proteome ID: UP000009294). However, the number of HSV-1-encoded proteins expressed by infected mDCs might differ from that of BHK21 cells.

Since the working group of Loret et al. (2008) reported the NEC components UL31 (NEC1) and UL34 (NEC2) to be absent in mature virions, one of the most surprising result of our mass spectrometry-based approach was the detection of the two NEC components UL31 and UL34 in L-particles derived from BHK21 cells (Figure 3), while exclusively UL34 was identified in L-particle preparations from mDCs (Figure 4). However, given the normalized signal intensity of UL31 to the mean of all glycoproteins, it appears that this protein is very low-abundant (<1%) in HSV-1-derived particles from BHK21 cells (Figure 3). In contrast, UL34 shows a higher normalized average signal intensity in H- and L-particles derived from BHK21 cells, while it is approximately 3.5 times overrepresented in L-particles compared to H-particles. Notably, L-particles derived from HSV-1-infected mDCs showed an equal normalized intensity of incorporated UL34 (Figure 4). In contrast to the findings of Loret et al. (2008), and in agreement with our observations, UL34 has been detected in virions of cytoplasmic extracts (Purves et al., 1992). Given the fact, that the production and function of non-infectious L-particles has not been intensively investigated yet, the reason for the higher abundance of UL34 in L-particles compared to H-particles from BHK21 cells requires further substantial investigation.

The HSV-1 tegument-associated proteins ICP0 and ICP4 are two key factors during HSV-1 replication. Both proteins are not only important modulators of intrinsic and innate immunity (Alandijany, 2019; Tognarelli et al., 2019), but are also crucial during the initiation of the viral gene expression cascade (Guo et al., 2010). On the one hand, it has been suggested that ICP4 might be an L-particle specific viral protein (Szilágyi and Cunningham, 1991; Heilingloh et al., 2015). On the other hand, several studies confirmed the presence of both viral proteins in mature virions (Yao and Courtney, 1989, 1992; Loret et al., 2008; Sedlackova and Rice, 2008; Delboy et al., 2010; Loret and Lippé, 2012; Russell et al., 2018). In line with the latter statement, our mass spectrometric data revealed ICP0 and ICP4 to be present in a comparable amount in L-particles and also in mature virions of HSV-1-infected BHK21 cells (Figure 3B). Based on their numerous functions during viral replication or immune evasion mechanisms, we suppose that these two viral proteins are not only virion- but also L-particle-associated components, to shape the microcellular environment for the benefit of the virus (Heilingloh et al., 2015).

Despite the hypothesis that H- and L-particles share a similar way of maturation, the non-infectious particles can also be produced in the absence of DNA replication and packaging (Dargan et al., 1995; Russell et al., 2018). Two important proteins regarding DNA replication are, e.g., ICP8, a single-stranded DNA binding protein (Zhou and Knipe, 2002; Weerasooriya et al., 2019), and the viral thymidine kinase UL23, which phosphorylates thymidine and thus prepares nucleotides for viral DNA synthesis (Chen et al., 1998; Pilger et al., 1999; Xie et al., 2019). Our mass spectrometric data of BHK21 cell-derived HSV-1 particles revealed ICP8 and UL23 to be present in L- and H-particles, representing the major hits among non-structural proteins (Table 2 and Figure 3). Interestingly, the normalized intensity of ICP8 and UL23 in L-particles derived from HSV-1-infected mDCs is extremely higher compared to particle preparations from HSV-1-infected BHK21 cells (Figures 3, 4). Since ICP8 is also described to inhibit stress granule formation and therefore prevents innate immune pathways, one possible reason for the presence of this viral protein might be the support of subsequent infection with HSV-1 mature virions and thus promoting viral replication and viral DNA transcription (Panas et al., 2015; Burgess and Mohr, 2018).

In L-particles derived from HSV-1-infected mDCs several proteins were detected counteracting cell death. First, ICP6, as major hit in mDC particles (Figure 4C), is an important viral protein for replication, since the ablation of ICP6 results in severe replication defects (Goldstein and Weller, 1988; Mostafa et al., 2018). While HSV-1 ICP6 activates programmed cell necrosis in mice (Huang et al., 2015), this viral protein acts as an inhibitor of cellular apoptosis and prevents the induction of necroptosis in human cells (Dufour et al., 2011; Guo et al., 2015; Yu et al., 2016; Mostafa et al., 2018). Particularly, the expression of both Ribonucleotide Reductase R1 subunits (ICP6 and ICP10) preserves from TNFα- and FasL-induced apoptosis (Dufour et al., 2011). Another protein leading to the blocking of apoptosis is gD, which is also one of the major hits in mDC-derived L-particles (Figure 4A; Zhou et al., 2000; Yu and He, 2016). Thus, it seems that HSV-1 L-particles transfer several proteins that manipulate host cell death to promote viral replication.

Upon an HSV-1 infection, anti-HSV-1 antibodies are generated during adaptive immune responses and are mainly targeted against the glycoproteins gD and gB (Criscuolo et al., 2019), two proteins which are highly expressed on the surface of HSV-1 virions (Cai et al., 1988; Eisenberg et al., 2012). Interestingly, L-particles derived from HSV-1-infected BHK21 cells and mDCs also contain significant amounts of both glycoproteins (Figures 3A, 4A). In Heptatitis B virus (HBV) infection, Rydell et al. showed that the presence of capsidless subviral particles diminished the neutralization capacity of anti-HBV antibodies (Rydell et al., 2017). Based on this observation, we suggest that L-particles released during an HSV-1 infection could also intercept antibodies targeted against gB or gD. This hypothesis is further supported by the finding that L-particles are predominantly produced by recently infected cells early after infection and to a lesser extent by cells located inside the center of infection (Alemañ et al., 2003) and thus might capture anti-HSV-1 antibodies prior to the release of infectious virions.

The functional role of L-particles during HSV-1 infection has been rarely investigated during the last years. Previous studies already revealed that infectious HSV-1 virions both hamper the mDC T cell stimulatory (Kruse et al., 2000; Pollara et al., 2003) as well as the iDC maturation capacity (Salio et al., 1999; Grosche et al., 2020). However, L-particles have not been analyzed regarding their impact on these two DC features. During DC maturation, L-particles specifically interfere with CD83 expression and not with any other analyzed maturation marker (Figure 7). By contrast to HSV-1-infected mDCs, which are completely inhibited in their T cell stimulatory capacity, L-particle-treated mDCs also show a significantly reduced ability to activate T cells (Figure 8). Based on these results, L-particles seem to disturb DC functions and therefore could be released to modulate surrounding bystander cells during HSV-1 infection.

In summary, the here presented comparative analysis extends recent reports regarding the viral protein composition of HSV-1-derived particles. In particular, we detected 63 viral proteins in infectious H- and L-particles from HSV-1-infected BHK21 cells and 41 viral proteins in L-particles from HSV-1-infected mDCs. Our data provide evidence that L-particles derived from HSV-1-infected BHK21 cells or mDCs transfer a plethora of viral proteins from infected cells to uninfected bystander cells. In this regard, we revealed that L-particles possess an immunomodulatory effect on DCs and suppress their T cell stimulatory capacity. Finally, we hypothesize that HSV-1 L-particles are produced and released by infected cells in order to shape and subvert the host’s immune response. L-particles not only foster HSV-1 replication via transferring essential viral proteins and modulate vital functions of DCs, but might also be essential for the interception of the host’s humoral antiviral immune response.

## Data Availability Statement

The MS proteomics data have been deposited to the ProteomeXchange Consortium via the PRIDE partner repository with the data set identifiers PXD020845 (BHK21 particles) and PXD020846 (mDC particles).

## Ethics Statement

The studies involving human participants were reviewed and approved by the “Ethik-Kommission der Friedrich-Alexander-Universität Erlangen-Nürnberg”. The patients patients/participants provided their written informed consent to participate in this study.

## Author Contributions

AB, LP, and AS designed the project and critically revised the manuscript. AB and PM-Z performed experiments. AB and LP interpreted the data. MK provided essential reagents and performed mass spectrometric measurements. JH performed PEAKS analyses of measurements from BHK21 cell- and mDC-derived particles. CH carried out electron microscopy. AB did formal the data analyses and prepared the original draft manuscript. All authors approved the final version of the manuscript.

## Conflict of Interest

The authors declare that the research was conducted in the absence of any commercial or financial relationships that could be construed as a potential conflict of interest.

## Abbreviations

BHK: baby hamster kidney
ICP: infected cell protein
RT: room temperature
UL: unique long
US: unique short.
